# Functional Outcomes After Imaging- and Orthopedic Test-Guided Evaluation of Shoulder Disorders: Systematic Review and Meta-Analysis

**DOI:** 10.3390/mps8060133

**Published:** 2025-11-03

**Authors:** Carlos Miquel García-de-Pereda-Notario, Luis Palomeque-Del-Cerro, Ricardo García-Mata, Luis Alfonso Arráez-Aybar

**Affiliations:** 1 Department of Anatomy and Embryology, Faculty of Medicine, Universidad Complutense de Madrid, 28040 Madrid, Spain; arraezla@med.ucm.es; 2 UCM Research Group No. 920202, Faculty of Medicine, Universidad Complutense de Madrid, 28040 Madrid, Spain; 3 Department of Physiotherapy, Faculty of Nursing and Physiotherapy “Salus Infirmorum”, Universidad Pontificia de Salamanca, 28015 Madrid, Spain; lpalomequede@upsa.es; 4 Escuela de Osteopatía de Madrid, 28033 Madrid, Spain; 5 Research Support, UCM IT Services, 28040 Madrid, Spain; rgarcia@ucm.es

**Keywords:** shoulder pain, rotator cuff injuries, diagnostic imaging, orthopedic tests, meta-analysis

## Abstract

Background: Shoulder soft tissue disorders, such as rotator cuff tears and subacromial impingement, are among the most common causes of musculoskeletal disability. Both physical examination tests and imaging techniques are routinely used in clinical settings; however, their respective contributions to patient outcomes and their potential complementarity remain underexplored. Methods: A systematic review and meta-analysis were conducted following PRISMA 2020 guidelines. Controlled clinical studies comparing pre- and post-intervention outcomes in adults with suspected or confirmed shoulder soft tissue pathology were included. Two groups were analyzed: studies using musculoskeletal imaging (ultrasound or MRI) and studies applying orthopedic physical examination tests (e.g., Neer, Hawkins, and Jobe). Functional outcomes were converted into standardized mean differences (SMDs) and synthesized using a random-effects model. Heterogeneity was quantified using the I^2^ statistic. Results: In total, 11 studies met the inclusion criteria (*n* = 6 imaging, *n* = 5 orthopedic tests). Imaging-based studies showed a pooled SMD of 4.85 (95% CI: 2.77–6.93), indicating substantial clinical improvement. Orthopedic test-based studies yielded a pooled SMD of 2.34 (95% CI: 1.27–3.41). Heterogeneity was high across both groups (I^2^ > 90%). Conclusions: Imaging was associated with a larger overall clinical effect, while orthopedic tests provided functional insight valuable for screening and monitoring. These findings support the complementary use of both strategies to enhance diagnostic accuracy and treatment planning in shoulder care.

## 1. Introduction

Shoulder pain is a prevalent musculoskeletal complaint, affecting up to 67% of individuals during their lifetime, and it ranks among the top three reasons for musculoskeletal consultations in primary care. The shoulder’s anatomical complexity and wide range of motion predispose it to various pathologies, particularly soft tissue disorders such as rotator cuff tears, subacromial impingement syndrome, and biceps tendinopathies. These conditions significantly impact patients’ quality of life and functional abilities [1].

Accurate diagnosis of shoulder pathologies is crucial for effective management. Traditionally, clinicians have relied on physical examination maneuvers—such as the Neer, Hawkins, Jobe, and Speed tests—to identify specific shoulder disorders. While these tests are cost-effective and easy to administer, their diagnostic accuracy is often limited by examiner variability and overlapping symptomatology. Conversely, imaging modalities like musculoskeletal ultrasound (MSK-US) and magnetic resonance imaging (MRI) offer detailed visualization of soft tissue structures, potentially enhancing diagnostic precision. However, imaging findings do not always correlate with clinical symptoms, and overreliance on imaging can lead to increased healthcare costs and potential overdiagnosis [2].

Despite the widespread use of both physical examination tests and imaging techniques, there is a lack of comprehensive analyses comparing their association with functional outcomes in shoulder disorders. Understanding the relative effectiveness of these diagnostic tools is essential for optimizing patient assessment and management strategies [3].

### 1.1. Purpose

This meta-analysis aims to evaluate the functional outcomes associated with the use of orthopedic physical examination tests and imaging modalities (MSK-US and MRI) in soft tissue shoulder disorders, specifically rotator cuff tears and subacromial impingement syndrome.

While these approaches assess different aspects of shoulder pathology—functional versus structural—this review aims to explore their respective contributions and potential complementarity in guiding patient management.

To date, no meta-analyses have been identified that directly assess the post-intervention functional impact associated with these diagnostic approaches. Given that the type of assessment may influence therapeutic decisions—whether surgical, rehabilitative, or pharmacological—it is essential to determine how each contributes to observed functional outcomes and whether their combination enhances clinical decision-making.

### 1.2. Hypothesis

We hypothesize that imaging modalities, particularly MSK-US and MRI, may be associated with greater average improvements in post-intervention functional outcomes, likely due to their ability to detect structural abnormalities that inform targeted treatment strategies.

However, this does not imply that one diagnostic method is inherently superior to the other. Rather, we propose that imaging and orthopedic physical examination tests offer distinct and complementary clinical information: the former provides anatomical and structural insights, while the latter captures functional impairments through real-time patient interaction.

This review aims to evaluate how each modality contributes to functional recovery and to determine whether their integration can enhance the diagnostic and therapeutic management of shoulder disorders.

## 2. Materials and Methods

### 2.1. Study Design

This study was conducted as a systematic review and meta-analysis in accordance with the Preferred Reporting Items for Systematic Reviews and Meta-Analyses (PRISMA) 2020 guidelines [4]. The protocol was registered in the International Prospective Register of Systematic Reviews (PROSPERO) prior to the literature search [Registration number: CRD420251083948].

The review was structured according to the PICO framework, as follows:

- Population: Adults with shoulder pain due to soft tissue injuries (e.g., rotator cuff tears and subacromial impingement).
- Intervention: Diagnostic approach using either orthopedic tests or imaging modalities.
- Comparison: Pre- vs. post-intervention measurements, or between different diagnostic strategies.
- Outcome: Functional clinical improvement, measured through validated quantitative scales.

Only studies reporting quantitative functional outcomes that could be converted into standardized mean differences (SMDs) were included in the meta-analysis component. This study was conducted as a systematic review and meta-analysis in accordance with the Preferred Re-porting Items for Systematic Reviews and Meta-Analyses (PRISMA) 2020 guidelines [4]. The protocol was registered in the International Prospective Register of Systematic Reviews (PROSPERO) prior to the lite-rature search [Registration number: CRD420251083948]. The review was structured according to the PICO framework, as follows: Only studies reporting quantitative functional outcomes that could be converted into standardized mean differences (SMDs) were included in the meta-analysis component.

The completed PRISMA 2020 checklist is provided in the Supplementary Materials.

### 2.2. Eligibility Criteria

Studies were included based on the following predefined inclusion criteria:

- Population: Adults aged 18 or older with or without clinical diagnosis of soft tissue shoulder disorders (e.g., rotator cuff tears, subacromial impingement, and tendinopathy).
- Study design: Controlled clinical studies (randomized or non-randomized) including at least two comparison groups (e.g., symptomatic vs. asymptomatic, pre- vs. post-intervention).
- Interventions: Studies evaluating either (a) imaging-based assessment strategies (MSK ultrasound, MRI, or radiography) or (b) orthopedic physical examination tests (e.g., Neer, Hawkins, and Jobe).
- Outcomes: Studies must report at least one of the functional outcome scores (ASES, CMS, SST, VAS, and WOSI) or other quantitative measures suitable for standardized mean difference (SMD) calculation.
- Accessibility: Only full-text, peer-reviewed, open access articles published in English or Spanish were included.
- Exclusion criteria:
- Case reports, reviews, editorials, conference abstracts, or animal studies.
- Studies evaluating only surgical techniques or therapeutic interventions without a pre–post functional assessment component.

Because the included primary studies seldom reported paired accuracy estimates (sensitivity/specificity at a common threshold) and instead provided continuous, validated functional scales before and after management, DTA-specific methods (PRISMA-DTA reporting, QUADAS-2 risk-of-bias, bivariate/HSROC, and threshold modeling) were not applicable. In line with our aim, we synthesized standardized mean differences (SMDs) of functional outcomes rather than accuracy parameters.

### 2.3. Information Sources and Search Strategy

A comprehensive literature search was conducted across four major electronic databases—PubMed, Scopus, ScienceDirect, and Web of Science—covering studies published between January 1997 and December 2024. The final search was completed in May 2025.

The search aimed to identify controlled studies investigating the functional outcomes of imaging techniques or orthopedic tests in shoulder disorders. Only those studies that reported quantitative functional outcome measures with sufficient data to calculate standardized mean differences (SMDs) were included in the meta-analysis component.

The search strategy combined Medical Subject Headings (MeSH) and free-text terms, encompassing the following domains:

- Anatomical regions (e.g., shoulder and glenohumeral joint);
- Target conditions (e.g., rotator cuff injuries and impingement syndromes);
- Diagnostic tools (e.g., orthopedic physical examination tests and imaging modalities);
- Functional outcome measures (e.g., validated clinical scores and quantitative scales).

The following key concepts and synonyms were used:

- Anatomical region: “Shoulder” and “Glenohumeral joint”;
- Target conditions: “Rotator cuff tear”, “Rotator cuff tendinopathy”, “Shoulder impingement syndrome”, “Subacromial syndrome”, and “Biceps tendinitis”;
- Diagnostic tools: “Physical examination”, “Orthopedic test”, “Special test”, “Clinical assessment”, “Jobe”, “Hawkins”, “Neer”, and “Speed test”;
- Imaging modalities: “Musculoskeletal ultrasound”, “Ultrasonography”, “Magnetic resonance imaging”, “MRI”, and “Radiography”;
- Outcomes: Although the search strategy included terms such as “Sensitivity”, “Specificity”, “Accuracy”, and “Likelihood ratio” to ensure broad coverage of diagnostic literature, only studies reporting functional outcomes through validated clinical scales—such as “ASES”, “CMS”, “VAS”, “SST”, or “WOSI”—were ultimately included in the meta-analysis.

An example of the search string used in PubMed was as follows:

((“Shoulder Injuries”[MeSH] OR “Rotator Cuff Injuries”[MeSH] OR “Shoulder Impingement Syndrome”[MeSH]) AND (“Diagnosis”[MeSH] OR “Physical Examination”[MeSH] OR “Diagnostic Imaging”[MeSH]) AND (“Sensitivity and Specificity”[MeSH] OR “Likelihood Functions” OR “ROC Curve” OR “Functional Outcome Assessment”)).

All references were imported into a reference manager (Zotero version 6.0.37) for duplicate removal and further screening.

### 2.4. Study Selection

**Figure 1:**
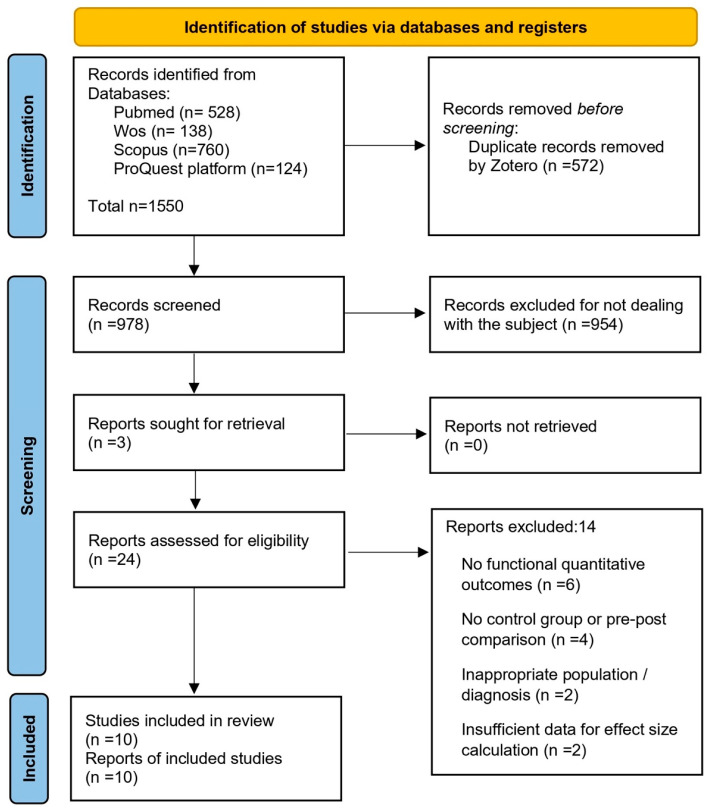
PRISMA flow diagram of the article selection process.

Two independent reviewers (blinded to each other’s decisions) screened the titles and abstracts for relevance. Full texts of potentially eligible studies were then reviewed to confirm inclusion. Disagreements were resolved by consensus or consultation with a third reviewer. The complete selection process is summarized in the PRISMA flow diagram (Figure 1).

### 2.5. Data Extraction

Data were extracted independently by two reviewers using a standardized form that included the following variables:

- Study identifiers (authors, year, and country);
- Population characteristics (sample size, age, and sex);
- Assessment tool evaluated (imaging or orthopedic test);
- Target pathology;
- Outcome measures related to functional improvement (e.g., validated clinical scales or quantitative scores);
- Functional scores (ASES, CMS, SST, VAS, etc.);
- Pre- and post-intervention values, when applicable.

Although some studies reported diagnostic metrics, only those providing quantitative functional outcomes with sufficient data to calculate standardized mean differences (SMDs) were included in the meta-analysis.

In cases where a single study included multiple valid comparison groups against a common control group, data from each group were extracted independently, provided that they met the eligibility criteria for inclusion in the meta-analysis.

If multiple publications derived from the same dataset were identified, the most comprehensive or recent version was prioritized for data extraction.

When a study reported more than one functional outcome scale, a single score was selected for meta-analytic purposes based on the following criteria: (1) availability of both mean and standard deviation values; (2) clinical relevance and validation of the scale in shoulder disorders; and (3) consistency with other included studies to enhance comparability. When multiple scales met these conditions, the most frequently reported outcome across the dataset (e.g., VAS, ASES) was prioritized. If relevant data were missing, corresponding authors were contacted for clarification.

Reporting of technical/operational details, such as anatomic region(s) imaged or examined, imaging acquisition parameters (e.g., MRI field strength, ultrasound transducer frequency), and operator experience/training, was not consistent across primary studies and could not be extracted for quantitative synthesis. Consequently, comparability between imaging findings and physical tests may be limited by unmeasured differences in the region of interest and system settings; this limitation is acknowledged in Section 4.

### 2.6. Data Synthesis and Statistical Analysis

Meta-analyses were conducted separately for studies evaluating imaging techniques and orthopedic tests. Functional outcomes were synthesized as standardized mean differences (SMDs) with 95% confidence intervals under a statistical random-effects model (DerSimonian–Laird), which accommodates both within- and between-study variance to yield conservative pooled estimates. When primary studies reported alternative effect-size metrics (e.g., raw mean differences, Cohen’s *d*), these were systematically converted into SMDs for methodological consistency and cross-scale comparability. Directionality was harmonized so that a higher SMD indicates improvement; scales where lower scores reflect better status (e.g., VAS) were inverted accordingly.

Between-study heterogeneity was quantified using Cochran’s Q and I^2^. I^2^, the proportion of total variability attributable to heterogeneity rather than sampling error, was computed as I^2^ = 100% × (Q − *df*)/Q, with *df* = *k* − 1 (number of studies minus one). Conventional thresholds of 25%, 50%, and 75% were interpreted as low, moderate, and high heterogeneity, respectively. Q(*k*) was reported to test whether observed dispersion exceeded chance expectations. These metrics were applied to continuous functional outcomes and were not intended for paired diagnostic-accuracy (sensitivity/specificity) data. Prespecified subgroup analyses compared imaging-based versus orthopedic test-based assessments.

All analyses were performed in Review Manager (RevMan) 5.4 and R (meta package). Because the included studies used diverse functional instruments (e.g., VAS, ASES, WOSI, and SST), standardization to SMD provided a coherent synthesis metric without assuming a single underlying true effect. The choice of a random-effects framework was further supported by the high heterogeneity observed (I^2^ > 90%), reflecting substantial methodological and clinical variability. Publication bias was not formally assessed because the number of studies per subgroup was below the accepted thresholds for reliable funnel-plot or Egger-type evaluations; this is acknowledged as a limitation [5,6].

## 3. Results

A total of 11 studies met the eligibility criteria and were included in the final analysis. Of these, six studies evaluated diagnostic imaging techniques (e.g., musculoskeletal ultrasound, MRI, and radiography), and five studies assessed orthopedic physical examination tests (e.g., Jobe, Neer, and Hawkins). These studies were conducted in a variety of clinical contexts and reported pre- and post-intervention functional outcomes using validated scales, including the Visual Analog Scale (VAS), the American Shoulder and Elbow Surgeons score (ASES), the Western Ontario Shoulder Instability Index (WOSI), the Simple Shoulder Test (SST), the Constant-Murley Score (CMS), and performance-based tests such as the Jobe test.

All included studies reported quantitative data sufficient for the calculation of standardized mean differences (SMDs), allowing their integration into the meta-analytic synthesis. Among the imaging studies, pooled effects ranged from modest (e.g., Brage et al. [7]: SMD = 1.31 [0.67, 1.96]) to extremely large (e.g., Paletta et al. [8], dataset C: SMD = 13.30 [9.98, 16.62]), reflecting substantial variability across samples and interventions. Notably, Kashef et al. [9] contributed two independent arms with large and consistent effects (SMD = 4.73 and 3.98), while Ashir et al. [10] also reported significant improvement (SMD = 2.24).

Regarding orthopedic tests, the effects were generally large and clinically meaningful. The most pronounced improvement was reported by MacDonald et al. [11] using the WOSI scale (SMD = 7.27 [5.38, 9.17]), followed by Caron et al. [12] (SMD = 2.75), Moezy et al. [13] using VAS (SMD = 2.24), and Jolles et al. [14] with the SST (SMD = 1.59). Moderate effects were observed in Struyf et al. [15] (SMD = 1.27), while Go and Lee [16] reported a smaller, statistically non-significant effect (SMD = 0.52 [−0.21, 1.25]).

**Figure 2:**
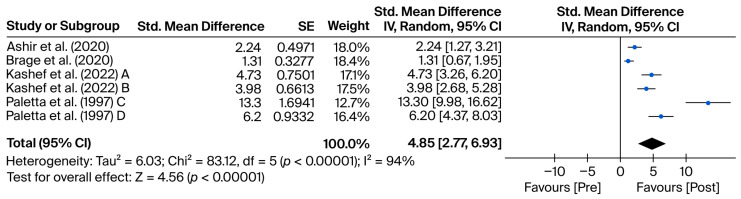
Forest plot for diagnostic imaging outcomes. Blue dots represent the standardized mean differences (SMDs) for individual studies, and the diamond indicates the pooled effect size. Data are derived from the studies by Brage et al. [7], Paletta et al. [8], Kashef et al. [9], and Ashir et al. [10].

**Figure 3:**
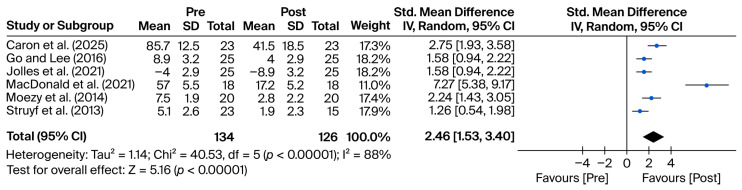
Forest plot for orthopedic test outcomes. Blue dots represent the standardized mean differences (SMDs) for individual studies, and the diamond indicates the pooled effect size. Data are derived from the studies by MacDonald et al. [11], Caron et al. [12], Moezy et al. [13], Jolles et al. [14], Struyf et al. [15], and Go and Lee [16].

Pooled meta-analytic results are presented separately for imaging techniques (Figure 2) and orthopedic tests (Figure 3). Table 1 summarizes the main characteristics and key findings of the studies included in the meta-analysis.

### 3.1. Diagnostic Tests

The forest plot (Figure 2) illustrates the results for diagnostic imaging studies, showing consistent improvement across all included studies. In all cases, the confidence intervals (CIs) are positive, with a pooled standardized mean difference (SMD) of 4.85 (95% CI: 2.77 to 6.93).

A high degree of heterogeneity was observed among the studies, with Q(5) = 83.12, *p* < 0.001, and an I^2^ value of 94%. This variability—likely due to differences in sample sizes, diagnostic tools, and functional outcome measures—justified the use of a random-effects model, assuming that each study estimates a distinct effect size. The pooled effect was statistically and clinically significant (Z = 4.56; *p* < 0.001), and the magnitude of the SMD indicates a substantial post-intervention improvement across all experimental groups.

In the study by Ref. [10], an SMD of 2.24 (95% CI: [1.27, 3.21]) was reported in favor of the post-intervention timepoint within the experimental group, based on a pre–post comparison of symptomatic tendons. To ensure consistency in the direction of effect, the original sign of the difference was inverted so that positive values would represent clinical improvement.

The study by Ref. [7] showed an SMD of 1.31 (95% CI: [0.67, 1.95]) in favor of the post-intervention measurement in the experimental group, also comparing pre- and post-intervention outcomes for symptomatic tendons. As with other included studies, the original result sign was reversed to standardize the direction of effect and ensure that positive values indicate clinical improvement.

In Kashef et al. [9] [A], an SMD of 4.73 (95% CI: [3.26, 6.20]) was observed in favor of the post-intervention timepoint in the experimental group, based on the comparison of pre- and post-intervention values in symptomatic tendons. The original sign was inverted to maintain a positive direction representing clinical improvement.

The study by Kashef et al. [9] [B] reported an SMD of 3.98 (95% CI: [2.68, 5.28]) in favor of the post-intervention timepoint in the experimental group, again based on a pre–post comparison. As in previous cases, the original sign of the result was reversed to ensure that positive values reflect clinical improvement.

In Paletta et al. [8] [C], an SMD of 13.30 (95% CI: [9.98, 16.62]) was obtained in favor of the post-intervention timepoint in the experimental group, comparing pre- and post-intervention values in symptomatic tendons. The direction of the effect was inverted to reflect a positive clinical outcome.

Finally, Paletta et al. [8] [D] reported an SMD of 6.20 (95% CI: [4.37, 8.03]) in favor of the post-intervention timepoint in the experimental group. As in the other included studies, the original sign was reversed to standardize interpretation and ensure that positive values represent clinical improvement.

### 3.2. Orthopedic Tests

The following forest plot (Figure 3) presents the pooled results of studies evaluating orthopedic physical examination tests. In all cases, clinical improvement was assessed by comparing pre- and post-intervention scores at the corresponding follow-up period of each study (ranging from weeks to months).

Heterogeneity was substantial, with Q(5) = 52.88, *p* < 0.001, and I^2^ = 91%, supporting the decision to use a random-effects model that accounts for both within- and between-study variability.

The overall combined effect indicates a statistically significant improvement, with a mean effect size of 2.34 (95% CI: [1.27, 3.41]), representing a clinically meaningful change (Z = 4.29; *p* < 0.001). All studies, except one, reported confidence intervals that did not cross the null value, indicating consistent improvements in clinical outcomes following interventions guided by orthopedic testing.

At the individual study level, considerable variability in effect sizes was observed: Ref. [11] reported the most pronounced change (SMD = 7.27 [5.38, 9.17]), reflecting a highly significant improvement. However, the unusually large magnitude warrants further examination of score scaling or potential data transformation issues.

Ref. [12] also showed a large effect size (SMD = 2.75 [1.93, 3.58]) on the WOSI scale, aligning with other findings using patient-reported functional outcomes. Ref. [14] found a substantial effect (SMD = 1.59 [0.89, 2.29]) using the SST scale.

Ref. [13] reported a significant and clinically relevant change on the VAS (SMD = 2.24 [1.43, 3.05]). Ref. [15] observed a moderate effect on the JOBE test (SMD = 1.27 [0.61, 1.94]), suggesting functional improvement in shoulder assessment.

Ref. [16] was the only study with a confidence interval crossing the null value (SMD = 0.52 [−0.21, 1.25]), indicating a non-significant or inconsistent change in CMS for this specific study.

## 4. Discussion

### 4.1. Summary of Main Findings

This meta-analysis included two distinct groups of studies: those that evaluated diagnostic imaging techniques, including musculoskeletal ultrasound (MSK-US) and magnetic resonance imaging (MRI), and those that evaluated the performance of orthopedic physical examination tests and associated functional clinical scales. The results revealed that both approaches demonstrated statistically and clinically significant improvements after intervention, although with different magnitudes of effect.

For the imaging group, the pooled effect size calculated using the standardized mean difference (SMD) was 4.85 (95% CI: [2.77, 6.93]), indicating a robust and clinically meaningful improvement in post-intervention values compared to baseline. This large effect size suggests a strong favorable clinical response to interventions guided by imaging findings. However, heterogeneity was very high (Q(5) = 83.12, *p* < 0.001; I^2^ = 94%), reflecting substantial variability among the included studies, likely due to differences in sample sizes, diagnostic methods used, and the variety of clinical scales applied [17].

In contrast, the studies using orthopedic tests also showed statistically significant improvement, with a pooled effect size of 2.34 (95% CI: [1.27, 3.41]). Although this effect was smaller than that observed in the imaging group, it remains clinically meaningful. Heterogeneity was also high in this group (Q(5) = 52.88, *p* < 0.001; I^2^ = 91%), again indicating notable variability in study methodology, outcome measures (e.g., WOSI, ASES, SST, and VAS), and participant characteristics [18].

Overall, these findings suggest that both imaging and orthopedic tests are valuable tools for evaluating and monitoring shoulder disorders, although they differ in their degree of diagnostic impact and precision. The high heterogeneity observed in both groups underscores the need to interpret the results with caution, considering the specific characteristics of each intervention, the tools applied, and the clinical context [19,20].

### 4.2. Comparison with Previous Studies

#### 4.2.1. Imaging Techniques

The results obtained in this meta-analysis, with a standardized mean difference (SMD) of 4.85 for imaging techniques, indicate a very strong clinical impact. This magnitude is consistent with previous studies that evaluated advanced imaging modalities, such as quantitative magnetic resonance imaging (MRI) and musculoskeletal ultrasound, in the assessment of rotator cuff disorders [11,12]. For instance, UTE-Cones-MT MRI has demonstrated significant alterations in macromolecular fraction (MMF) and T2 relaxation time in patients with rotator cuff tendinopathy compared to asymptomatic individuals, highlighting the potential of imaging biomarkers to detect early tendon degeneration [13].

Similarly, musculoskeletal ultrasound has shown not only diagnostic utility but also clinical applicability in monitoring patient outcomes. Previous trials have reported significant improvements in functional scales (VAS and QuickDASH) following ultrasound-guided interventions in subacromial syndrome, reinforcing its role as a tool for both diagnosis and follow-up [14]. Importantly, all variables analyzed in the included studies were quantitative, enabling the calculation of means and standard deviations and subsequent transformation into standardized mean differences (SMDs). To standardize interpretation, scales were inverted when necessary, so that all positive values consistently represented clinical improvement (e.g., reductions in VAS or QuickDASH scores).

These findings align with systematic evidence indicating the high diagnostic accuracy of ultrasound and MRI for characterizing rotator cuff pathology [12]. Notably, Roy et al. [21] confirmed equivalent sensitivity and specificity for ultrasound, MRI, and MR arthrography in detecting partial and full-thickness rotator cuff tears, underscoring the robustness of imaging modalities when performed with standardized protocols and experienced operators. Collectively, these data strengthen the rationale for incorporating musculoskeletal ultrasound and MRI as reliable tools for early detection and clinical monitoring in shoulder disorders.

#### 4.2.2. Discussion of Orthopedic Tests

In the orthopedic tests group, the meta-analysis yielded a pooled standardized mean difference (SMD) of 2.34 (95% CI: 1.27–3.41), indicating a clinically significant effect, though of lower magnitude compared to imaging. These results are in agreement with clinical studies emphasizing the value of functional tests and standardized scales in the evaluation and follow-up of shoulder pain [15].

For example, rehabilitation programs focused on scapular motor control have shown clinically relevant effects, with significant improvements in the SDQ scale (Cohen’s d = 0.93) and reductions in pain during orthopedic tests such as Neer, Hawkins, and Empty Can, with effect sizes ranging from 0.76 to 1.04 [16]. These results highlight that orthopedic tests are not only diagnostic but also sensitive to functional changes during rehabilitation, particularly in cases of scapular dyskinesis.

Other investigations have demonstrated significant improvements in postural parameters and ultrasound findings after scapular stabilization programs, supporting the ability of orthopedic tests to reflect structural and functional adaptations when integrated with complementary tools [17]. In addition, studies comparing clinical scales (ASES, SST, and Constant) with kinematic outcomes derived from inertial sensors found strong correlations (e.g., r = 0.80 for SST and power score) [16,18], confirming their reliability as objective indicators of functional status.

Taken together, these studies validate the use of orthopedic tests as sensitive and clinically relevant tools for both diagnosis and outcome monitoring [19]. Their integration with validated scales and targeted therapeutic interventions enhances their applicability in daily clinical practice, and as outlined in APTA guidelines, they represent a cornerstone of standardized functional assessment in patients with shoulder pain [22].

### 4.3. Clinical Implications

The results of this meta-analysis support the clinical utility of both imaging techniques and orthopedic tests in the diagnostic and therapeutic management of shoulder disorders. The standardized mean difference (SMD) was higher in the imaging group (4.85), although orthopedic tests also showed a clinically significant effect (SMD = 2.34).

From a practical perspective, these findings endorse the use of quantitative functional scales, such as ASES, SST, Constant, and QuickDASH, not only for initial diagnosis but also for monitoring patient progress throughout treatment. The ability to calculate means and standard deviations enabled effect standardization across studies, and reversing the direction of scales when necessary ensured consistent interpretation of clinical improvement [22].

Moreover, the fact that both diagnostic strategies showed clinical impact supports their integration into combined protocols. In clinical practice, orthopedic tests provide an accessible and cost-effective first-line functional assessment, while imaging techniques allow for confirmation of specific rotator cuff lesions, tissue damage assessment, and objective evaluation of treatment response [23].

Overall, the findings of this meta-analysis suggest that a dual assessment strategy, combining structural and functional evaluation, may optimize diagnostic and therapeutic processes in shoulder care, enhancing both precision and longitudinal follow-up in real-world clinical settings [24,25].

These findings reinforce the importance of adopting a complementary diagnostic strategy. Rather than positioning one method as superior, clinicians should consider the strengths of each: imaging provides anatomical detail that can support precise diagnosis, while orthopedic tests offer practical, low-cost assessment of functional impairment. Their integration may enhance decision-making and improve patient outcomes in a resource-sensitive manner.

### 4.4. Integration with Current Clinical Guidelines

The findings of this meta-analysis are consistent with, and extend, the recommendations of current international guidelines. The European Society of Musculoskeletal Radiology (ESSR) has emphasized the role of musculoskeletal ultrasound as a first-line imaging tool in the evaluation of rotator cuff disorders, highlighting its diagnostic accuracy, safety, and accessibility compared to magnetic resonance imaging (MRI) [26]. Moreover, the ESSR consensus on image-guided interventional procedures for the shoulder further supports the utility of ultrasound not only for diagnosis but also for therapeutic monitoring and guidance [27]. These recommendations align with our results, which demonstrated the strong clinical impact of imaging techniques, particularly when standardized protocols were applied.

Similarly, the American Physical Therapy Association (APTA) has provided guidelines supporting the use of validated orthopedic tests and functional scales in the clinical evaluation of shoulder pain. The APTA clinical practice guidelines highlight the relevance of tools such as the ASES, SST, and Constant scores for assessing functional limitations and guiding treatment strategies [22]. In addition, systematic reviews have reinforced the diagnostic accuracy of both ultrasound and MRI in the characterization of rotator cuff disorders [21], consistent with our findings showing that both imaging and orthopedic tests contribute clinically meaningful information.

Despite these alignments, an important gap remains: current guidelines typically address imaging and orthopedic tests separately, with limited integration of both strategies into combined diagnostic pathways. Our meta-analysis addresses this gap by providing pooled evidence that supports the complementary value of structural imaging and functional testing. These results underscore the need for future updates of clinical guidelines to explicitly recommend integrated approaches, combining imaging modalities and orthopedic evaluations, in order to optimize diagnostic accuracy and therapeutic decision-making in patients with shoulder disorders.

### 4.5. Strengths and Limitations of the Study

One of the main strengths of this meta-analysis is the clear stratification of the studies included into two distinct groups, which allowed for accurate comparison of the clinical impact of imaging techniques and orthopedic tests in shoulder disorders.

A comprehensive search across PubMed, Scopus, ScienceDirect, and Web of Science confirmed this gap, thereby justifying the present systematic review and meta-analysis.

This thematic structure adds both clinical and methodological value, enabling specific conclusions for different diagnostic tool categories.

Another important strength is the exclusive use of quantitative variables (mean and standard deviation), which allowed for standardized and robust SMD calculations. Additionally, the normalization of outcome direction, by inverting scores where a decrease indicated improvement, ensured coherent interpretation of combined effects. This methodological approach enhances comparability across studies with diverse clinical designs.

It should also be noted that all included studies incorporated both experimental and control groups, which reinforces the internal validity of the observed effects and reduces intervention bias.

However, this meta-analysis also presents certain limitations. First, statistical heterogeneity was high in both groups (I^2^ > 90%), indicating considerable variability among studies in terms of population, interventions, clinical scales, and follow-up duration. Although this heterogeneity is common in clinical reviews, it limits the generalizability of pooled results [20].

Second, some studies used different functional scales (VAS, ASES, SST, SDQ, and QuickDASH) to assess clinical outcomes, which, even though corrected through standardization, could introduce bias due to differences in scale sensitivity [28].

In addition, no formal publication bias analysis or quality assessment (e.g., using QUADAS-2 or PEDro scale) was performed. Incorporating such assessments in future studies could help strengthen the evidence.

Lastly, the inclusion of studies with relatively small sample sizes may have affected the precision of effect estimates and the width of confidence intervals.

In summary, although the results show clinically meaningful and consistent effects, they should be interpreted with caution, considering the methodological and population variability across the included studies.

### 4.6. Future Research and Clinical Conclusions

The findings of this meta-analysis highlight the clinical relevance of integrating both imaging techniques and orthopedic tests into the diagnostic approach for shoulder disorders. However, several unanswered questions remain, warranting further investigation.

First, future comparative clinical studies should aim to combine both imaging and functional assessments in longitudinal designs to better explore their relationship, complementarity, and role in therapeutic decision-making. It would be particularly valuable to implement protocols that combine musculoskeletal ultrasound with validated functional scales both before and after treatment, enabling simultaneous structural and functional monitoring [29].

Second, more methodologically consistent studies are needed, ideally using standardized clinical instruments such as ASES, SST, or QuickDASH, to facilitate future comparisons and meta-analyses. Long-term follow-up should also be included to assess the stability of clinical outcomes [30].

Additionally, future research should include cost-effectiveness analyses in real-world clinical settings to determine the feasibility of routinely implementing advanced imaging techniques compared to standard clinical evaluations. In this regard, the accessibility and low cost of orthopedic tests continue to position them as a valuable first-line screening tool [31].

Finally, future studies are encouraged to incorporate objective digital tools, such as inertial sensors or biomechanical analysis platforms, to complement subjective scales and enhance diagnostic precision and reproducibility, particularly in active young populations or athletes [32].

In summary, this meta-analysis shows that both imaging and orthopedic tests provide valuable information, albeit from different diagnostic perspectives. Their combined use, supported by high-quality studies, may optimize the diagnosis and follow-up of shoulder pathologies, leading to more precise and personalized interventions [19].

## 5. Conclusions

This meta-analysis has demonstrated that both diagnostic imaging techniques and orthopedic tests are associated with clinically significant functional improvements in the evaluation of shoulder disorders, although they operate through different mechanisms and with varying magnitudes of effect.

While imaging modalities, particularly musculoskeletal ultrasound and MRI, showed a higher pooled standardized mean difference in reported outcomes, this should not be interpreted as evidence of diagnostic superiority. Imaging provides detailed structural information that can support treatment planning, whereas orthopedic tests offer functional insights that are immediately accessible, low-cost, and valuable for both diagnosis and monitoring.

These findings support the complementary use of structural and functional diagnostic strategies, which may enhance both the diagnostic process and treatment outcomes for patients with rotator cuff injuries and other shoulder dysfunctions. They may also guide clinicians and researchers in the development of integrated, evidence-based diagnostic protocols tailored to the resources available in each clinical setting.

Future studies should focus on evaluating this integration through controlled longitudinal designs, incorporating standardized functional scales and reproducible imaging techniques, as well as new objective digital tools to improve diagnostic accuracy and outcome tracking.

## Figures and Tables

**Table 1:** Summary of included studies—diagnostic imaging.

Author (Year)	Country	Design	Sample (*n*)	Clinical Management Context	Duration	Outcomes	Key Findings (Conclusions)
Reference [9]	Egypt	Randomized Controlled Trial (RCT)	*n* = 45 (15 per group)	Scapular exercises + kinesiotaping or ultrasound vs. control	4 weeks	VAS, QuickDASH, strength, ROM	Kinesiotaping showed greater improvements in pain and function compared to ultrasound and control.
Reference [8]	USA	Longitudinal observational study	*n* = 33 (18 instability, 15 RCT)	Radiographic evaluation before and after surgical repair (capsulolabral or rotator cuff)	2 years	Glenohumeral and scapulothoracic kinematics	Most patients showed normalized glenohumeral kinematics after surgery; scapulothoracic kinematics normalized only in the rotator cuff group.
Reference [10]	Pakistan	Prospective quantitative imaging study	*n* = 48 shoulders (24 patients)	Quantitative MRI evaluation (UTE-Cones-MT and T2 mapping)	Single session	MMF, T2, WORC, VAS	Symptomatic tendons showed lower MMF and higher T2 values compared to controls. Alterations also present in asymptomatic contralateral shoulders.
Reference [7]	Spain	Prospective cohort study	*n* = 23	12-week exercise program for supraspinatus tendinopathy	12 weeks	Strain elastography (RAW, DELT), DASH, VAS, tendon thickness, MRI	Improvements in pain and function (DASH, VAS); no structural changes observed on ultrasound or MRI.
Reference [15]	Belgium	Randomized Clinical Trial (RCT)	*n* = 46 (23 experimental, 23 control)	Scapula-focused treatment (education + scapular control exercises + manual therapy) vs. conventional treatment (mobility and strengthening exercises)	6 weeks	SPADI, pain (VAS), rotator strength, functional tests	The scapular group showed significantly greater improvements in pain, function, and SPADI compared to the control group after 6 weeks of treatment.
Reference [13]	Iran	Randomized Clinical Trial (RCT)	*n* = 68 (34 experimental, 34 control)	Scapular stabilization exercises vs. conventional exercises for subacromial impingement	12 weeks	VAS, abduction, external rotation, head posture, scapular symmetry	Significant improvements in VAS, joint mobility, and posture in the experimental group compared to the control.
Reference [16]	South Korea	Randomized Clinical Trial (RCT)	*n* = 30 (15 experimental, 15 control)	Scapular stability exercises vs. standard office routine	6 weeks	Scapular stability, ultrasound imaging, posture	Significant reduction in forward head posture and greater scapular stability in the intervention group.
Reference [11]	Canada	Prospective longitudinal study	*n* = 40 (20 per group)	Specific conservative rehabilitation	12 months	WOSI, ASES, SST	Progressive improvements in WOSI, ASES, and SST after 12 months of intervention.
Reference [14]	Switzerland	Observational study	*n* = 65 (34 experimental, 31 control)	No specific comparative intervention	3–12 months	ASES, SST	Increase in SST; no significant changes in ASES.
Reference [12]	Canada	Controlled clinical trial	*n* = 60 (30 per group)	Conservative treatment in patients with shoulder instability vs. control	24 months	WOSI, ASES	Both groups improved in WOSI and ASES, with no significant differences between groups.

## Data Availability

All data analyzed in this study were extracted from previously published articles, which are publicly accessible. The complete dataset used for the meta-analysis is available from the corresponding author upon reasonable request.

## Supplementary Materials

The following supporting information can be downloaded at: https://www.mdpi.com/article/10.3390/mps8060133/s1, PRISMA 2020 Checklist.
