# Supplementary material for: Functional Outcomes After Imaging- and Orthopedic Test-Guided Evaluation of Shoulder Disorders: Systematic Review and Meta-Analysis
Source: Methods Protoc. 2025 Nov 3;8(6):133. doi: 10.3390/mps8060133 (PMC12641838; doi:10.3390/mps8060133)
Supplement: Supplementary file 1 [file mps-08-00133-s001.zip › mps-3751736-supplementary.pdf]

PRISMA 2020 Checklist

Section and Topic	Item #	Checklist item	Location where item is reported
**TITLE**			
Title	1	Identify the report as a systematic review.	Title (first page)
**ABSTRACT**			
Abstract	2	See the PRISMA 2020 for Abstracts checklist.	Abstract (first page)
**INTRODUCTION**			
Rationale	3	Describe the rationale for the review in the context of existing knowledge.	Section 1. Introduction (párr. 1 y 2)
Objectives	4	Provide an explicit statement of the objective(s) or question(s) the review addresses.	Section 1.1 Purpose
**METHODS**			
Eligibility criteria	5	Specify the inclusion and exclusion criteria for the review and how studies were grouped for the syntheses.	Section 2.2 Eligibility Criteria
Information sources	6	Specify all databases, registers, websites, organisations, reference lists and other sources searched or consulted to identify studies. Specify the date when each source was last searched or consulted.	Section 2.3 Information Sources and Section Strategy
Search strategy	7	Present the full search strategies for all databases, registers and websites, including any filters and limits used.	Section 2.3: includes MeSH terms and a PubMed search. Very well described.
Selection process	8	Specify the methods used to decide whether a study met the inclusion criteria of the review, including how many reviewers screened each record and each report retrieved, whether they worked independently, and if applicable, details of automation tools used in the process.	Section 2.4 Study Selection
Data collection process	9	Specify the methods used to collect data from reports, including how many reviewers collected data from each report, whether they worked independently, any processes for obtaining or confirming data from study investigators, and if applicable, details of automation tools used in the process.	Section 2.5 Data Extraction
Data items	10a	List and define all outcomes for which data were sought. Specify whether all results that were compatible with each outcome domain in each study were sought (e.g. for all measures, time points, analyses), and if not, the methods used to decide which results to collect.	Section 2.5 (last paragraph)
	10b	List and define all other variables for which data were sought (e.g. participant and intervention characteristics, funding sources). Describe any assumptions made about any missing or unclear information.	Section 2.5 (paragraph 2 y 3)
Study risk of bias assessment	11	Specify the methods used to assess risk of bias in the included studies, including details of the tool(s) used, how many reviewers assessed each study and whether they worked independently, and if applicable, details of automation tools used in the process.	No risk of bias assessment performed
Effect measures	12	Specify for each outcome the effect measure(s) (e.g. risk ratio, mean difference) used in the synthesis or presentation of results.	Section 2.6 Data Synthesis and Statistical Analysis
Synthesis methods	13a	Describe the processes used to decide which studies were eligible for each synthesis (e.g. tabulating the study intervention characteristics and comparing against the planned groups for each synthesis (item #5)).	Section 2.6 and end of 2.5: the studies are grouped into two categories (imaging vs. physical tests), according to predefined criteria.
	13b	Describe any methods required to prepare the data for presentation or synthesis, such as handling of missing summary statistics, or data conversions.	Section 2.5 (last paragraph) and 2.6: conversion to SMD; the sign of some

Section and Topic	Item #	Checklist item	Location where item is reported
			scales was inverted to standardize the direction of the effect.
	13c	Describe any methods used to tabulate or visually display results of individual studies and syntheses.	Section 2.6 and Section 3 (Figures 2 and 3; Tables 1 and 3): use of forest plots and summary tables.
	13d	Describe any methods used to synthesize results and provide a rationale for the choice(s). If meta-analysis was performed, describe the model(s), method(s) to identify the presence and extent of statistical heterogeneity, and software package(s) used.	Section 2.6: random-effects model (DerSimonian–Laird), heterogeneity assessed with I^2, use of RevMan 5.4 and R (meta package).
	13e	Describe any methods used to explore possible causes of heterogeneity among study results (e.g. subgroup analysis, meta-regression).	Section 2.6 and 4.1–4.2: the use of a random-effects model is justified due to the high heterogeneity (I^2 > 90%). No meta-regression or formal subgroup analyses were performed, but sources of variability are discussed.
	13f	Describe any sensitivity analyses conducted to assess robustness of the synthesized results.	A sensitivity analysis was conducted by excluding the studies with extreme effect sizes (e.g., Paletta et al., 1997), with minimal impact on the overall pooled estimates, suggesting robustness of the findings.
Reporting bias assessment	14	Describe any methods used to assess risk of bias due to missing results in a synthesis (arising from reporting biases).	Due to the small number of studies included in each synthesis (n < 10), a formal assessment of publication bias (e.g., funnel plot or Egger's test) was not performed, following current Cochrane methodological guidance [Higgins JPT, Thomas J, Chandler J, et al. (Eds.) Cochrane Handbook for Systematic Reviews of Interventions Version 6.4 (updated February 2023). Cochrane; 2023. Available from: https://training.cochrane.org/handbook].
Certainty assessment	15	Describe any methods used to assess certainty (or confidence) in the body of evidence for an outcome.	Certainty of the evidence was not formally assessed using GRADE due to heterogeneity in study design and outcomes. Future updates may consider applying this approach.

PRISMA 2020 Checklist

Section and Topic	Item #	Checklist item	Location where item is reported
**RESULTS**			
Study selection	16a	Describe the results of the search and selection process, from the number of records identified in the search to the number of studies included in the review, ideally using a flow diagram.	Section 3. Results (initial paragraph). In addition, Figure 1 (PRISMA Flow Diagram) is included.
	16b	Cite studies that might appear to meet the inclusion criteria, but which were excluded, and explain why they were excluded.	A list of excluded full-text articles with reasons for exclusion is available upon request.
Study characteristics	17	Cite each included study and present its characteristics.	Tables 2 and 3 summarize authors, countries, design, intervention, duration, and key findings.
Risk of bias in studies	18	Present assessments of risk of bias for each included study.	No risk of bias assessment was performed
Results of individual studies	19	For all outcomes, present, for each study: (a) summary statistics for each group (where appropriate) and (b) an effect estimate and its precision (e.g. confidence/credible interval), ideally using structured tables or plots.	Table 1, Tables 2 and 3, and in the forest plots (Figures 2 and 3).
Results of syntheses	20a	For each synthesis, briefly summarise the characteristics and risk of bias among contributing studies.	Results section (Table 1; study characteristics summarized). No risk of bias assessment was performed.
	20b	Present results of all statistical syntheses conducted. If meta-analysis was done, present for each the summary estimate and its precision (e.g. confidence/credible interval) and measures of statistical heterogeneity. If comparing groups, describe the direction of the effect.	Section 3.1 y 3.2
	20c	Present results of all investigations of possible causes of heterogeneity among study results.	Not done — no investigation of heterogeneity causes was performed because subgroup analysis was considered unnecessary due to small sample size.
	20d	Present results of all sensitivity analyses conducted to assess the robustness of the synthesized results.	Not done. We excluded those studies with high bias, and we have a small sample size because there is limited literature available.
Reporting biases	21	Present assessments of risk of bias due to missing results (arising from reporting biases) for each synthesis assessed.	No assessment of reporting bias was performed

PRISMA 2020 Checklist

Section and Topic	Item #	Checklist item	Location where item is reported
Certainty of evidence	22	Present assessments of certainty (or confidence) in the body of evidence for each outcome assessed.	No assessment of certainty of evidence was performed
**DISCUSSION**			
Discussion	23a	Provide a general interpretation of the results in the context of other evidence.	Sections 4.1 and 4.2: the imaging and orthopedic test results are interpreted and compared with previous literature (references 11–19).
	23b	Discuss any limitations of the evidence included in the review.	Section 4.4: heterogeneity, variability in clinical scales, and small sample sizes are discussed.
	23c	Discuss any limitations of the review processes used.	Not discussed
	23d	Discuss implications of the results for practice, policy, and future research.	Section 4.3 y 4.5
**OTHER INFORMATION**			
Registration and protocol	24a	Provide registration information for the review, including register name and registration number, or state that the review was not registered.	Methods (2.1) The protocol was registered in the International Prospective Register of Systematic Reviews (PROSPERO) prior to the literature search. [Registration number: CRD420251083948].
	24b	Indicate where the review protocol can be accessed, or state that a protocol was not prepared.	Methods (2.1)
	24c	Describe and explain any amendments to information provided at registration or in the protocol.	Methods (2.1)
Support	25	Describe sources of financial or non-financial support for the review, and the role of the funders or sponsors in the review.	End of manuscript, after Conclusions (Funding section)
Competing interests	26	Declare any competing interests of review authors.	End of manuscript, after Conclusions (Funding section)
Availability of data, code and other materials	27	Report which of the following are publicly available and where they can be found: template data collection forms; data extracted from included studies; data used for all analyses; analytic code; any other materials used in the review.	All data extracted from included studies are available upon reasonable request to the corresponding author. No analytical code or software scripts were used beyond standard functions of

PRISMA 2020 Checklist

Section and Topic	Item #	Checklist item	Location where item is reported
			RevMan 5.4 and R meta package

From: Page MJ, McKenzie JE, Bossuyt PM, Boutron I, Hoffmann TC, Mulrow CD, et al. The PRISMA 2020 statement: an updated guideline for reporting systematic reviews. BMJ 2021;372:n71. doi: 10.1136/bmj.n71.
This work is licensed under CC BY 4.0. To view a copy of this license, visit https://creativecommons.org/licenses/by/4.0/
